# Behavioural and oceanographic isolation of an island-based jellyfish (*Copula sivickisi*, Class Cubozoa) population

**DOI:** 10.1038/s41598-021-89755-7

**Published:** 2021-05-13

**Authors:** Jodie A. Schlaefer, Eric Wolanski, Jonathan Lambrechts, Michael J. Kingsford

**Affiliations:** 1 Research Hub for Coral Reef Ecosystem Functions, James Cook University, Townsville, QLD 4811 Australia; 2 College of Science and Engineering, James Cook University, Townsville, QLD 4811 Australia; 3 ARC Centre of Excellence for Coral Reef Studies, James Cook University, Townsville, QLD 4811 Australia; 4 TropWATER, James Cook University, Townsville, QLD 4811 Australia; 5 Institute of Mechanics, Materials and Civil Engineering, Université de Louvain, 1348 Louvain-La-Neuve, Belgium

**Keywords:** Animal behaviour, Biooceanography, Population dynamics

## Abstract

Cubozoan jellyfish are classified as plankton despite the strong swimming and orientation abilities of cubomedusae. How these capabilities could affect cubozoan population structures is poorly understood. Medusae of the cubozoan *Copula sivickisi* can uniquely attach to surfaces with the sticky pads on their bells. Biophysical modelling was used to investigate the spatial scales of connectivity in a *C. sivickisi* population. When the medusae were active at night they could maintain their observed distribution on fringing reef if they attached to the reef when the current speed exceeded a moderate threshold. This behaviour facilitated the isolation of a *C. sivickisi* population on reefs fringing Magnetic Island, Queensland, Australia. Within this distribution, there was considerable within bay retention and medusae rarely travelled > 3 km. The few (< 0.1%) medusae lost from the island habitat were largely advected into open water and away from the mainland coast which lies 8 km from the island. Given that successful emigration is unlikely, the island population probably represents a stock that is ecologically distinct from any mainland populations. The cosmopolitan distribution of *C. sivickisi* could contain incipient or cryptic species given the small scales of connectivity demonstrated here.

## Introduction

The dynamics of populations are underpinned by their spatial structures. The geographic ranges of marine species are generally inhabited by one or multiple metapopulations composed of mesopopulations/stocks which are largely self-contained^1,2^. Stocks may be further divided into connected local populations^1^. Currents and/or swimming can transport individuals’ long distances, connecting distant population units^3^. In contrast, species may inhabit systems with retentive currents and/or may limit dispersion through biological mechanisms^4,5^.

Information on how box jellyfish (class Cubozoa) populations are spatially structured is generally lacking. Most cubozoans inhabit highly structured nearshore estuarine and/or coastal habitats (e.g., mangroves, and rocky and coral reefs;^6^). Despite being classified as plankton, cubomedusae must navigate through these structured ecosystems, and they have associated sophisticated behaviours^5,7–9^ and strong swimming abilities^5,9–11^. Further, estuarine and coastal habitats often have areas/zones of reduced currents, and hence increased retention. For example, current speeds tend to decrease with proximity to a solid boundary (current shear;^12,13^), and boundaries are prevalent in estuarine (e.g., the bottom or side of an estuarine channel) and coastal (e.g., the top of a submerged reef) systems. Additionally, complex, retentive eddy fields can be generated as currents flow past/through structures such as headlands, mangrove prop roots and coral reef matrices (the ‘sticky water effect’;^14^). Cubozoan stocks may form at relatively small spatial scales within estuarine and coastal systems given the orientation and swimming capabilities of cubomedusae, and the availability of refuges from strong current within structured estuarine and coastal habitats. Indeed, small (hundreds of metres to kilometres) to medium (tens to hundreds of kilometres) spatial scales of separation have been identified among *Chironex fleckeri* stocks^5,15,16^.

The data required to assess population structures at fine spatial scales are lacking for most cubozoan species. The *Copula* *sivickisi* metapopulation covers tropical and temperate latitudes in the Pacific, Indian and Atlantic Oceans^6,17–19^. While this expansive distribution suggests broad dispersal and connectivity, *C. sivickisi* are seemingly adapted to limit dispersion. The exposure of *C. sivickisi* to dispersive currents is limited by their reproductive mode^20,21^ and by the behaviour of their strong swimming medusae^9,21,22^. *C. sivickisi* medusae have sticky pads on the apex of their bells^21^ which they use to attach to substrates when they are inactive (e.g., at night^22^), or to avoid being displaced by currents^9^.

Schlaefer et al.^9^ predominantly found *C. sivickisi* medusae in shallow water in association with *Sargassum sp.* algae and hard coral. Their distribution was determined to be largely restricted to bands of reef which spanned 4 km along two small bays at Magnetic Island, Queensland, Australia. Magnetic Island lies 8 km from the mainland. Given the observed restricted distribution and the poor dispersal potential of *C. sivickisi*, it was predicted that the Magnetic Island population was isolated from any mainland *C. sivickisi* populations. However, stock differentiation techniques have not been applied at a sufficiently small scale (i.e., hundreds of meters to kilometres) to test this prediction.

Few stock differentiation techniques have been applied to *C. sivickisi*. Numerous techniques have been used to successfully differentiate stocks at relatively small spatial scales in other jellyfish species including: demographics^23^, genetics^24^, biogeochemistry^15^, morphometrics^16^ and biophysical modelling^5^. Biophysical models integrate representations of both the physical and biological components of model systems. For instance, combined hydrodynamic and behavioural models can simulate the movements of organisms in aquatic environments, considering both complex flows and species properties and behaviours. These models can provide high-definition information on the scales separating stocks of aquatic species and they are particularly useful when the behaviour of the focal organism has the potential to greatly influence its dispersal, which is true for *C. sivickisi* medusae. Uniquely, biophysical models can be applied to elucidate the mechanisms of stock separation by systematically varying the model parameters to assess their influence on dispersal.

The objective of this study was to determine the structure of the *C. sivickisi* population inhabiting Magnetic Island. Specifically, we aimed to: (1) map the extent of the population with underwater Jellyfish Camera units (JCams;^9^), (2) apply biophysical models to explore the role of *C. sivickisi* medusae behaviour in restricting their distribution to shallow reefs, and (3) expand the modelling to ascertain the internal structure of the Magnetic Island population and to (4) determine if the island population represented a stock that was isolated from any mainland populations.

## Materials and methods

### Mapping the population

**Figure 1:**
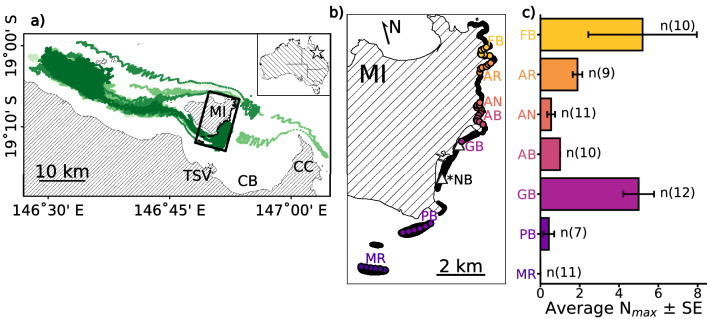
The *Copula sivickisi* population at Magnetic Island (MI) in the Townsville (TSV) region (star), Queensland, Australia. (**a**) Population structure analysis (green colour scale). The simulated export of *C. sivickisi* from MI, i.e., the tracks of the *C. sivickisi* medusae lost from MI reefs as adults during the 2017 season (< 0.1% of seeded medusae). The different colours distinguish the results of the five replicate model runs. *CB* cleveland bay, *CC*  cape cleveland. The rectangle over MI shows the extent of pane (**b**). (**b**) Population mapping (yellow to purple colour scale). The JCam survey design covering Middle Reef (MR), Picnic Bay (PB), Geoffrey Bay (GB), Alma Bay (AB), Alma North (AN), Arthur Bay (AR) and Florence Bay (FB). The dots show where the JCams were deployed, and are colored by location. The white triangles show the sites in Nelly Bay (NB; * no JCams were deployed in NB) and GB where the modelled currents were extracted for Fig. 2. Land is filled with a hatch pattern and reefs are shown in black. (**c**) The abundance (average N_max_ from JCams) of *C. sivickisi* medusae at each of the colorcoded locations. The number of sites averaged per location are indicated; total n = 70.

The geographic extent of the *Copula sivickisi* population inhabiting the east coast of Magnetic Island was mapped in the 2017 medusae season (September to November) with underwater Jellyfish Camera units (JCams; Fig. 1b;^9^). The photopositive *C. sivickisi* medusae were attracted to the light on each JCam and recorded by the adjacent camera in 30-min deployments. Following established methods for managing potential repeat counts^25^, the abundance of medusae was measured by counting the maximum number of medusae in any single frame of video during the deployment period (Nmax).

The east coast is comprised of numerous adjacent bays which each contain shallow fringing reefs. The distribution of *C. sivickisi* medusae has been found to overlap with the islands reefal habitat bands which are abundant in *Sargassum sp.* algae and coral^9^. JCams were, therefore, deployed at sites on reefal habitat in the east coast bays: Picnic Bay (6 sites), Geoffrey Bay (2 sites), Alma Bay (6 sites), Alma north (6 sites), Arthur Bay (6 sites) and Florence Bay (6 sites). Further, Middle Reef lies approximately 3 km to the south west of Magnetic Island, between the island and the mainland and could potentially act as a bridge between the Magnetic Island *C. sivickisi* population and any mainland populations. JCams were also deployed at 6 sites on Middle Reef to explore this possibility. The coordinates of the sites were pre-determined from Google Earth satellite images to guide the night sampling. Darkened patches in the images were assumed to correspond to moderate to high reefal habitat availability (i.e., > 33% substrate coverage by *Sargassum sp.* and coral) following^9^, and the presence of reefal habitat at the sites was later confirmed from the JCam footage. Each bay/reef, excluding Geoffrey Bay, was sampled two times over six non-consecutive nights from the 25th of September to the 30th of October. Two sites within Geoffrey Bay, randomly placed near the *C. sivickisi* hotspot identified in^9^, were sampled on each of the 6 trips to confirm the presence of *C. sivickisi* medusae throughout the sampling period. Geoffrey Bay was sampled at a lower spatial resolution compared to the other bays/reefs (2 sites compared to 6 sites) because we could be confident in detecting medusae in Geoffrey Bay given the information derived from the comprehensive sampling of the bay in previous medusae seasons (presented in^9^). Further, a higher temporal resolution (6 trips to Geoffrey Bay compared to two for the other bays/reefs) was required to verify that *C. sivickisi* were present during the entire sampling period, and this had associated logistical constraints such as the weather.

### Biophysical modelling

#### Hydrodynamic description

The two-dimensional version of the Second-generation Louvain-la-Neuve Ice-ocean Model [SLIM; 26] was used to model the currents off the eastern coast of Magnetic Island and in the surrounding region. A detailed description of SLIM and the specifics of its application in this study is presented in the supplementary information. Magnetic Island lies in the central area of the Great Barrier Reef (GBR). The currents in the GBR system are largely driven by the jets of the South Equatorial Current (SEC) which flow westward across the Coral Sea and collide with the outer reefs of the GBR^27^. In the central GBR, the North Caledonian Jet (NCJ) from the SEC generally diverges around the Queensland Plateau before meeting the outer reefs^27^. In addition to these regional scale forcings, the waters within the GBR system are shallow (mostly < 60 m) so winds can force currents at a local scale^28^. The model domain extended westward into the Coral Sea (Fig. S1a). The northern boundary of the model was approximately set in the middle of the Queensland Plateau, above the latitude where the southern divergence of the NCJ typically meets the GBR. The southern extent of the domain was far to the south of Magnetic Island to avoid confounding errors from the open boundary forcing. The unstructured SLIM grid was made coarser in open water and finer near coasts and over reefs. This reduced the number of elements required to effectively capture both the regional scale currents and the small-scale currents near complex bathymetry where there was horizontal current shear. The sea surface elevation simulated in SLIM was validated against a local tide gauge and the simulated currents were validated against current meters at 4 sites at or near Magnetic Island (Figs. S1b, S3, Table S1). The hydrodynamic fields were simulated every 3 min, and saved every 15 min. This high temporal resolution was used to match the fine spatial resolution of the SLIM grid to ensure that complex coastal and reefal hydrodynamic features were effectively simulated. The dispersion of medusae was simulated by coupling the hydrodynamic output with models of *C. sivickisi* medusae behaviour.

#### *C. sivickisi* medusae behaviour

Two candidate models (base and dependent; Table S2) of the behaviour of *C. sivickisi* medusae were developed from the results of Schlaefer et al.^9^ and from data sourced from the literature^12,13,22^. In both models, the behaviour of medusae changed with position in relation to reefal habitat (on/off) and with time of day (day/night; Table 1). Medusae ‘on habitat’ interacted with the habitat, while medusae ‘off habitat’ had no habitat associated behavioural cues. The *C. sivickisi* medusae were programmed to be nocturnal, i.e., they were inactive during the day and active at night. The positions of the model medusae were re-assigned every 3 min.

**Table 1:** Descriptions of the base and dependent models of *Copula Sivickisi* medusae behaviour.

	Day (07:00–18:57)	Night (19:00–06:57)	
On habitat	**Base = dependent** Attached to habitat	**Base** Near bottom. Unattached with cue to swim horizontally to the centre of the habitat band	**Dependent** **Current speed < cut off:** Near bottom. Unattached with cue to swim horizontally to the centre of the habitat band **Current speed ≥ cut off:** Attached to habitat
Off habitat	**Base = dependent** Near Bottom. Unattached with no horizontal swimming cues	**Base = dependent** Near bottom. Unattached with no horizontal swimming cues	

The behaviour of medusae in the base model was determined by the time of day (day or night) and the location of medusae in relation to reefal habitat. An additional behaviour was added to the dependent model where medusae only swam at night if the current speed at their location was less than a set cut off. When medusae swam, their speed was set to their critical swim speed (U_crit_; 4.9 cm s^−1^) or their sustainable swim speed (U_sust_; 2.45 cm s^−1^).

The two candidate models only differed by the on habitat, night time behaviour. In the base model, medusae on habitat at night were made to swim toward the habitat midline regardless of the current speed. A current speed dependent attachment behaviour was added to the dependent model. A dependent model medusa on habitat at night would only swim if the current speed at its position was less than a set cut off. If the current speed equalled or exceeded the cut off, the medusa would attach itself to the habitat, thereby becoming immovable.

#### Role of behaviour in retention

Biophysical modelling was used to investigate the role of medusae behaviour in maintaining their observed distribution in Nelly Bay and Geoffrey Bay of Magnetic Island, where they were predominantly found on shallow bands of fringing reef habitat (see^9^). Base and dependent model runs were performed. When the medusae swam in both models, their speed was set to their calculated critical swim speed (U_crit_; 4.9 cm s^−1^;^9^) i.e., the maximum swim speed *C. sivickisi* medusae can maintain for an extended period of time^29^. The current speed cut off for the dependent model medusae was set to 6 cm s^−1^ (^9^, Table S2). Medusae were additionally modelled as passive in a control scenario to determine the level of retention without behaviour.

*C. sivickisi* medusae were released from bands of reefal habitat in Nelly Bay and Geoffrey Bay (15 locations spaced at 200 m intervals, Fig. S1c) on a date at the end of September, near the start of the 2016 *C. sivickisi* season (Table S3). The runs ended after 30 days, approximately the length of one lunar cycle, allowing for the assessment of retention under spring and neap tides. No mortality was included. Five replicate runs were performed per candidate model/parameterisation. The number of medusae remaining in the bays was counted through time, with the outer limit of the Nelly and Geoffrey Bay catch zones set to the deepest cross shelf and furthest longshore boundaries of the bays reefal habitat bands (Fig. S1c). The catch zones represented the limits of the distribution of *C. sivickisi* medusae in the bays as measured by^9^.

#### Role of behaviour in retention sensitivity analysis

A sensitivity analysis was performed to examine how changing the dependent behavioural model parameterisation effected the simulated retention of *C. sivickisi* medusae on reefal habitat. Specifically, the retention of *C. sivickisi* medusae on reefal habitat in Nelly Bay and Geoffrey Bay, Magnetic Island, was re-simulated with the dependent model populated with different swim speeds and a range of current speed cut off values. The other model specifications were kept constant and matched the role of behaviour in retention analysis. Two medusae swim speeds were tested. In one set of runs the swim speed was set to U_crit_^9^, as in the main analysis. There was considerable variability in the U_crit_ for *C. sivickisi* medusae calculated by^9^, U_crit_ = 4.9 cm s^−1^ ± 4.4 standard deviation. Therefore, U_crit_ was halved in the second set of runs to more conservatively model the swimming capabilities of *C. sivickisi* medusae. Halving U_crit_ gave an estimate of the medusae’s sustainable swim speed (i.e., the maximum speed they could sustain without signs of fatigue^30^; U_sust_ = 2.45 cm s^−1^). Five different attachment to habitat current speed cut offs (3, 4.5, 6, 7.5 and 9 cm s^−1^) were modelled to cover and extend the range of current speeds at which *C. sivickisi* medusae attached to the tank in the swim trials conducted by^9^ (Table S2). The two swim speeds were each combined with all cut off’s of greater speed. The cut-off speeds had to be greater otherwise the model medusae would never be active in currents faster than they could swim against, so they would physically never be advected from the virtual reefal habitat. Eight dependent model parameterisations were, therefore, tested in the sensitivity analysis (U_crit_ with cut offs 6, 7.5 and 9 cm s^−1^, and U_sust_ with all cut offs). Note, the parameterisation with the swim speed set to U_crit_ and the cut off set to 6 cm s^−1^ was the dependent model parameterisation presented in the main role of behaviour in retention analysis. The number of *C. sivickisi* medusae in Nelly Bay and Geoffrey Bay was counted through time as in the primary role of behaviour in retention analysis. The range of current speeds with the potential to expatriate medusae from the virtual habitat was calculated as the speed difference between the medusae swim speed and the set current speed cut-off at which medusae attached to the habitat. The effect of the size of the expatriating current range (explanatory variable) on the percentage of model medusae remaining in the bays at the end of the 30 day model runs (response variable) was tested with a regression analysis (python 3.7.4, statsmodels 0.11.0, Fig. S6).

#### Population structure

Additional modelling runs were performed to elucidate the spatial structure of the *C. sivickisi* population on Magnetic Island. The dependent model was used because the dependent model behavioural suite was more biologically realistic. This was affirmed by the results of the role of behaviour in retention analysis and the sensitivity analysis. The high retention of *C. sivickisi* medusae on the reefal habitat in Nelly Bay and Geoffrey Bay simulated in the dependent model runs matched the measured restriction of *C. sivickisi* to the bays narrow reefs^9^. Further, high within-bay retention was simulated under numerous dependent model setups, so the selection of any one of these parameterisations for use in the population structure analysis would give biologically realistic results (see results section ‘sensitivity analysis’).

The spatial and temporal extent of the modelling was scaled up for the population structure analysis, and this had associated computational costs. The dependent model was, therefore, parameterised with a medusae swim speed of U_crit_, and a current speed cut off of 6 cm s^−1^ for the population structure analysis. In addition to simulating biologically realistic retention, this parameterisation had relatively low between replicate variability. Consequently, fewer model medusae were needed to get consistency between the population structure replicates (see results section ‘population structure’), which is indicative of a robust outcome^31^.

The population structure modelling was informed by the JCam population mapping. Specifically, the mapping revealed that *C. sivickisi* medusae were present all along the east coast of Magnetic Island, from Picnic Bay to Florence Bay (see results section ‘Population extent’), and confirmed the presence of suitable habitat (shallow reefs with *Sargassum sp.* and coral) in all the sampled bays and Middle Reef. Model *C. sivickisi* medusae were, therefore, released across the extent of the mapped east coast distribution. Further, as part of assessing the potential for Middle Reef to act as a bridge between the island and mainland populations, medusae were also seeded from Middle Reef despite their absence from the reef in the JCam survey. Medusae were, therefore, released from 32 locations spaced every 500–600 m along the near continuous reefal habitat band which runs from Middle Reef to Florence Bay (Fig. S1d; Table S4). The movements of the model medusae were simulated over an entire medusae season (September to November 2017; Table S4). Mortality was included in the model as an exponential decay function to simulate the natural attrition of medusae (Fig. S5, Table S4). The curve approached but would never reach 100% mortality, and the small percentage of medusae remaining after 54 days (near maximum age) were killed (3.2% of released medusae). Five replicate model runs were performed.

A measure of relative connectivity was generated to assess the internal structure (i.e., levels of self-seeding and inter bay/reefal connectivity) of the Magnetic Island *C. sivickisi* population (aim 3). The ‘on habitat’ zones in the behavioural models were divided into 32 detection zones, around the 32 seed locations. The detection zones had an average area of 0.08 km^2^ ± 4 × 10^–3^ (range 0.04–0.13 km^2^). Notably, because the detection zones were approximately evenly distributed in space, larger bays/reefs had more detection zones than smaller bays/reefs. The instances of unique connections (from source seed location to sink detection zone) made by adult medusae within and between zones were counted throughout the season. Medusae were considered adults 25 days post release because *C. sivickisi* medusae > 5 mm in diameter (half their maximum size^6^), are generally sexually mature^20^, and *C. sivickisi* medusae would take around 25 days to grow to 5 mm [unpublished data]. To generate the measure of relative connectivity, a log base 10 transformation was performed on the counts + 1 data, and the transformed data was scaled from 0 (no connections) to 1 (most connections).

The potential for connectivity between the island and mainland populations, and thereby the potential for Magnetic Island to represent an isolated stock (aims 4), was assessed by tracking the positions of all adult medusae lost from habitat at/near Magnetic Island through time. The combined trajectories showed the maximum extent of the emigration plume from the Magnetic Island population.

### Ethics approval

This work was supported by the ARC Centre of Excellence for Coral Reef Studies and the Australian Lions Foundation. It was conducted in accordance with James Cook University’s Animal Ethics Committee’s policies, procedures and guidelines. No specific permissions were required.

## Results

### Population extent

The population of *Copula sivickisi* medusae extended along the entire east coast of Magnetic Island (Fig. 1c). The reefal habitat on the east coast was dominated by *Sargassum sp.* algae and coral, and near continuous patches of reef were separated by small areas of sand. The abundance of medusae peaked in Geoffrey Bay, mid-way along the east coast, and Florence Bay, the northern most sampling location. Medusae were 2 to 5 times more abundant at Geoffrey Bay and Florence Bay compared to the other sampled locations on the east coast. *C. sivickisi* medusae were always absent from Middle Reef where the habitat was similarly dominated by *Sargassum sp.* and coral.

### Role of behaviour in retention

**Figure 2:**
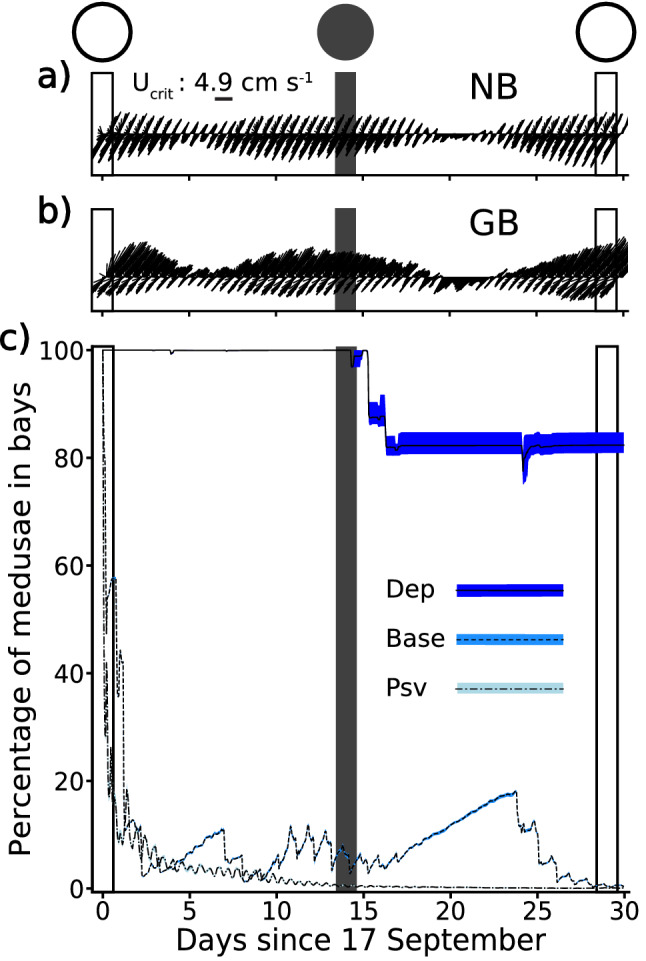
Role of behaviour in retention main analysis (blue colour scale). The simulated currents experienced by medusae (i.e., half the depth averaged current) at sites in (**a**) Nelly Bay (NB) and (**b**) Geoffrey Bay (GB), as marked in Fig. 1. The lengths of the sticks indicate the speed and the sticks are oriented in the direction the current flowed to. The reference stick shows the stick length for a speed of U_crit_. (**c**) The average percentage of *Copula sivickisi* medusae remaining in NB and GB through time as simulated with the passive (Psv), base (Base) and dependent (Dep) models. The bands underlying each line indicate the range of percentages simulated among the five replicate model runs. The circles at the top of the figure, and the vertical lines running down from them, show when there was a full (white) or new (grey) moon.

Biologically realistic retention was only simulated when the model medusae’s behavioural set included the diel and current speed dependent attachment behaviour (Fig. 2c). Most (95.1% ± 0.1) of the medusae from the passive model runs were advected from Nelly Bay and Geoffrey Bay within four days of their release (Fig. 2c), when there were strong currents associated with a spring tide (Fig. 2a,b). A majority of the passive medusae left in the bays were expatriated through time, with near zero remaining within the two bays toward the end of the 30-day model run.

*C. sivickisi* medusae intermittently maintained positions on reefal habitat when they were programmed with the base behavioural set (swam at night irrespective of the current speed; Fig. 2c). During the initial four-day period of strong currents around a spring tide, the base model medusae were lost at a similar rate (95.8% ± 0.1) to the passive medusae. There were periods during the 30-day runs when the number of base medusae in the bays fluctuated in diel cycles (e.g., from the 9th to 16th day after release, in the moderate currents between a neap and a spring tide) or steadily increased (e.g., from the 16th to the 23rd day after release, when there were weak currents associated with a neap tide) in reaction to the strength of the currents (Fig. 2a,b). Despite these periods, the percentages of base model medusae retained in the bays approached zero at the end of the simulation period, like in the passive runs.

The *C. sivickisi* medusae consistently maintained positions on habitat in the more biologically realistic dependent model runs (Fig. 2c). The retention simulated by the dependent model was decoupled from the change in current speeds that occurred in association with the lunar cycle. No dependent model medusae were lost during the initial period of strong currents. Medusae with the dependent attachment behaviour could only be expatriated from reefal habitat by currents faster than their swim speed, but slower than the current speed cut off (4.9 cm s^−1^ < current speed < 6 cm s^−1^), accounting for the relative stability in the percentage of dependent model medusae remaining in the bays through time. There were only two substantial expatriation events during the 30 day runs. Nearly 18% of the medusae initially seeded in the bays were lost in the consecutive events which occurred between the 15th day and 17th day after release. The medusae remaining in the bays after these events were retained for the duration of the runs, with an average percentage of 82.4% ± 0.7 remaining in the bays at the end of the runs.

#### Sensitivity analysis

**Figure 3:**
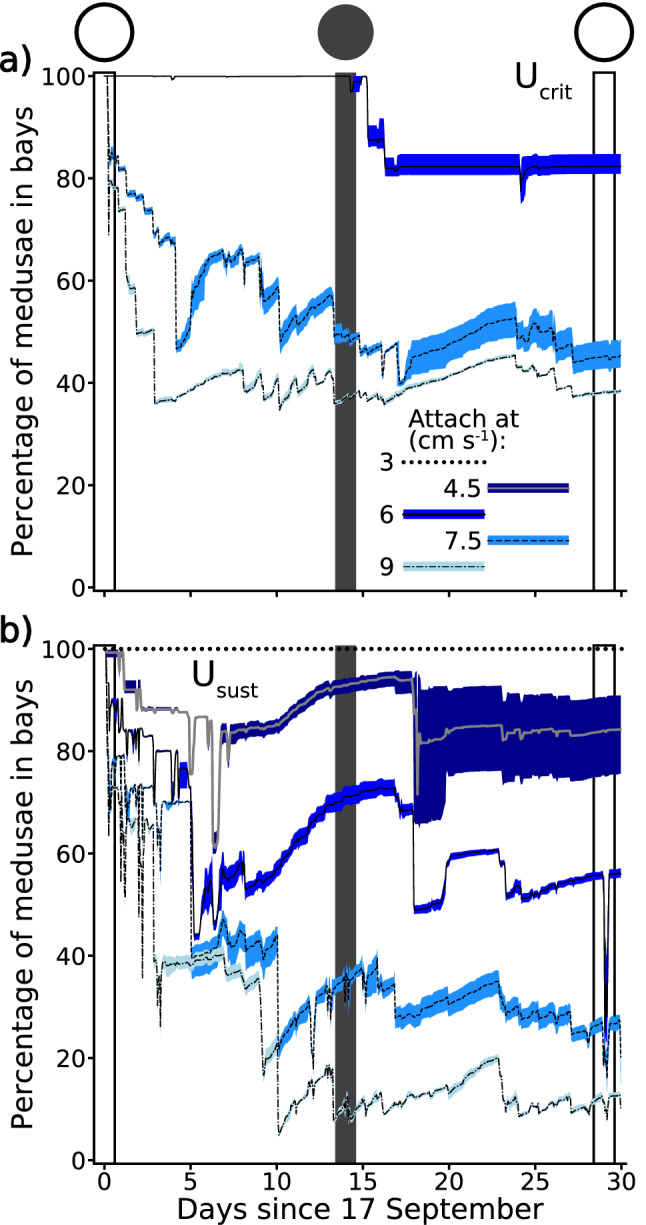
Role of behaviour in retention sensitivity analysis (blue colour scale). The average percentage of *Copula sivickisi* medusae remaining in Nelly Bay and Geoffrey Bay through time as simulated with the different dependent model parameterisations. Model medusae were programmed to swim at either (**a**) U_crit_ or (**b**) U_sust_, and to attach to habitat at current speed cut offs of 3, 4.5, 6, 7.5 and 9 cm s^−1^. The bands underlying each line indicate the range of percentages simulated among the five replicate model runs. The circles at the top of the figure, and the vertical lines running down from them, show when there was a full (white) or new (grey) moon.

The percentage of *C. sivickisi* medusae remaining in Nelly Bay and Geoffrey Bay after 30 days did not approach zero in any of the dependent model parameterisations (Fig. 3). Moderate to high retention of medusae was simulated under several parameterisations, including scenarios where medusae swam at U_sust_ (from 56.0% ± 0.3 to 100% ± 0.0 remaining at attachment cut offs ≤ 6 cm s^−1^, Fig. 3b), and scenarios where they swam at the faster U_crit_ (from 38.4% ± 0.2 to 82.4% ± 0.7 remaining at cut offs ≥ 6 cm s^−1^, Fig. 3a).

The exact level of simulated retention was sensitive to changes in the medusae swim speed and in the current speed cut off when medusae stopped swimming and attached to habitat. Greater retention of *C. sivickisi* medusae in the bays was recorded in the dependent model parameterisations with the higher swim speed. For example, in the pair of parameterisations where medusae attached at current speeds ≥ 9 cm s^−1^, the retention nearly quadrupled when the swim speed was increased from U_sust_ to U_crit_. The retention also increased with decreases in the attachment speed cut off. For example, in the set of parameterisations where the medusae swam at U_sust_, an average of over 5 times more medusae were retained at the end of the 30-days when they attached to habitat at current speed cut offs ≥ 6 cm s^−1^ compared to when they attached at speeds ≥ 9 cm s^−1^.

Notably, the range of current speeds with the potential to expatriate medusae (current speeds greater than the swim speed, but less than the attachment cut off) narrowed with increases in the medusae’s swim speed and decreases in the attachment cut off. There was a strong negative relationship between the range size and the retention at the end of the model run, where retention decreased as the range increased (Fig. S6). This relationship explained 91% of the variability in the end of scenario retention simulated among the different parameterisations.

### Population structure

**Figure 4:**
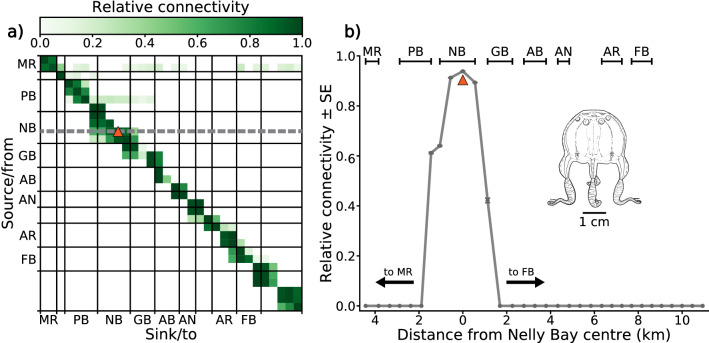
Population structure analysis (green colour scale). Substructure of the *Copula sivickisi* population at Magnetic Island. (**a**) The relative connectivity between source/from and sink/to detection zones over the entire 2017 medusae season. Each pixel represents one detection zone, and zones have been pooled by reefs/bays (solid lines in the matrix). The matrix shows the result from one of the five replicate model runs. (**b**) A cross section of the connectivity matrix, showing the average relative connectivity ± SE (n = 5) with distance from a focal detection zone in the centre of Nelly Bay. The cross section has been taken along the grey dashed line in pane (**a**), and the focal detection zone where source = sink is indicated by the orange triangles in panes (**a**) and (**b**). The geographic locations of the reefs/bays are shown in Fig. 1. The insert in pane (**b**) shows a male *C. sivickisi* medusae.

The simulated population of *C. sivickisi* on Magnetic Island was well mixed. The in-zone retention rate was high, as indicated by the high relative connectivity between corresponding source and sink locations (top left to bottom right diagonal in matrix; Fig. 4a). Inter bay connections were made between adjacent bays, but over small distances (range: 400 m to 3.7 km). For example, medusae released from central Nelly Bay were recorded in Geoffrey Bay as adults, in a detection zone 1.1 km to the north east of their initial release location (Fig. 4b). Adult medusae from central Nelly Bay were also recorded 1.4 km to the south west of their release location, in a detection zone in Picnic Bay.

Middle Reef did not act as a steppingstone between the simulated island population and any mainland populations. The simulated medusae were rarely exported north-eastward from Middle Reef to Magnetic Island reefs (Fig. 4a, Fig. S7). Export in the opposite direction, south-westward from the island to Middle Reef, was almost non-existent.

Further, no direct emigration of *C. sivickisi* medusae from the Magnetic Island population to any mainland populations was predicted. The vast majority of *C. sivickisi* medusae maintained positions on reefal habitat fringing Magnetic Island. An average of only 1163.6 ± 24.2 SE adult medusae (< 0.1% of medusae seeded from Magnetic Island) were lost from the reefs fringing Magnetic Island. No adult medusae were transported from Magnetic Island reefs to the mainland coast (Fig. 1a). Medusae lost from habitat tended to be advected to the north east, travelling maximum distances of < 50 km from Magnetic Island. A few medusae were expatriated to the east south east, and they were transported smaller distances (< 25 km from Magnetic Island).

## Discussion

Model *Copula sivickisi* medusae maintained their restricted distribution on fringing reef habitat by limiting their exposure to dispersive currents. Further modelling revealed that the Magnetic Island population of *C. sivickisi* likely represents a stock given the improbability of ecologically significant numbers of *C. sivickisi* medusae successfully emigrating from the Island to the mainland.

### The importance of behaviour in retention

The behaviour of *C. sivickisi* medusae was critical to maintaining their restricted distribution on fringing reef habitat, in alignment with the predictions of^9^. High retention was only simulated in the biophysical model when medusae swimming near reefs at night were programmed to attach to the reef when the current exceeded 6 cm s^−1^ (i.e., the dependent model). The modelled behaviour is biologically realistic. *C. sivickisi* medusae are nocturnal and when subjected to swim trials they stuck to the sides of the chamber to avoid being pushed back by the flow^9^. Further, *C. sivickisi* medusae exhibited a strong preference for reefal habitat in habitat choice experiments^9,22^. Other aquatic animals have similarly adapted behaviours to control their movements within environments with dispersive currents, and the behaviours likewise have major consequences for population structure (e.g., selective tidal stream transport^32^ and vertical migration^33^).

### Sources of error

The behaviours of *C. sivickisi* medusae were modelled with a high level of sophistication. However, models invariably only capture a portion of the variability that exists in natural systems (e.g., ontogenetic, among individual and stochastic variation^34^). For example, constant average swim speeds and constant attachment to habitat current speed cut offs were included in this study’s biophysical model. The variability surrounding these constants, from sources such as medusae varying their swim speed and attachment behaviour in reaction to external factors (e.g., currents, the presence of prey) and differences in individuals swimming abilities, was not incorporated into the model. Incorporating more natural variability could potentially improve the accuracy of the modelled outcomes.

The conclusions derived from the modelled outcomes were robust to changes in the behavioural model parameterisation. The moderate to high retention simulated by most of the trialled dependent model parameterisations in the sensitivity analysis supports this deduction. While the conclusions drawn were robust, the exact modelled level of retention of *C. sivickisi* medusae on reefal habitat was sensitive to changes in the model parameterisation. Specifically, retention increased with an increase in the swim speed and a reduction in the attachment cut off. Such sensitivities are inherent in modelling systems, and have similarly been reported in other biophysical modelling studies (e.g.,^5^).

### Population structure

The population of *C. sivickisi* medusae at Magnetic Island was found to extend at least as far as the range of the JCam survey on the island which covered the entire east coast. The population probably extends further to the north west beyond the limits of the survey. The preferred habitat of *C. sivickisi* medusae is fringing reef and the presence of reefs is, therefore, a prerequisite for the presence of *C. sivickisi*^9^. There is probably fringing reef on the northern face of the island. However, fringing reef is rare on the west coast where the dominant habitats are shallow sediment flats and seagrass beds^35^.

The Magnetic Island population of *C. sivickisi* is probably a robust ecologically, and likely genetically, distinct stock. It is unlikely that significant numbers of *C. sivickisi* medusae from the population on the east coast of Magnetic Island could successfully emigrate to the mainland. There was no evidence that Middle Reef acts as a ‘stepping stone’^36^ between island and mainland *C. sivickisi* populations. *C. sivickisi* medusae were absent from Middle Reef despite the presence of their preferred habitat (*Sargassum sp.* and coral^9^), and their ability to physically maintain positions on the reef. This suggests their absence could be driven by ecological factors, and there are numerous possibilities. For example, in the model, the medusae were unlikely to disperse from Magnetic Island to Middle Reef; they maintained positions on reefal habitat and rarely traversed the sand separating Middle Reef from the island. If some few medusae made it to Middle Reef they may be at greater risk of local extinction due to the greater exposure of Middle Reef compared to the sheltered bays where *C. sivickisi* medusae were present. The different geographies could have substantially different pelagic (i.e., plankton prey^37^ and fish predator^38^) communities. Further, no medusae in the model directly emigrated from the east coast of Magnetic Island to the mainland. It is exceptionally unlikely that medusae lost from the unmapped north would be transported south/south west to the mainland given the net currents in the 2017 medusae season. In this year, the net currents transported the majority of medusae that were lost from the east coast to the northwest, parallel to the mainland and not toward it. Severe storms such as tropical cyclones can drastically increase water turbulence (e.g.,^39^), and they could, therefore, increase dispersal distances. However, the *C. sivickisi* medusae season at Magnetic Island (September to November) lies mostly outside of the cyclone season (November to May). Further, the turbulence of a severe storm event could physically damage the gelatinous bodies of *C. sivickisi* medusae^40^ and the fresh water input following a storm could impair the medusae as cubomedusae are sensitive to low salinities^41^. Additionally, medusae would probably be more vulnerable to predation in the open water of the crossing, away from the refuge of the structured reefal habitat^42^. The isolation of the Magnetic Island stock suggests that genetically distinct *C. sivickisi* stocks may commonly be differentiable at surprisingly small spatial scales.

We found limited potential for medusae from a Magnetic Island source population to emigrate to a mainland sink; however, alternate connectivity hypotheses were not explored. For example, the export of *C. sivickisi* medusae could occur in the opposite direction, from a mainland source to an island sink. However, this alternate hypothesis is implausible for two reasons: (1) the prevailing direction of current transport is longshore, and (2) the mainland coastal habitat in the vicinity of Magnetic Island is primarily sandy beaches and mud flats which are unsuitable for *C. sivickisi* habitation. Schaefer et al.^9^ also proposed a different alternate connectivity hypothesis that the ‘sticky’ earlier life stages of *C. sivickisi* (i.e., the polyps, embryo sacs and planula larvae^21^) could disperse by remaining attached to the shedded sporophytes of *Sargassum sp.* algae. To survive, the *C. sivickisi* would critically need to metamorphose into medusae before the *Sargassum sp.* washes onshore and rots, and the medusae would need to find suitable habitat. Similar mechanisms of connectivity have been described for other marine species. For example, floating objects from Ecuador and/or Peru with assemblages of juvenile and adult reef fish have run aground on Gorgona Island hundreds to thousands of kilometres away^43^.

There is a growing body of evidence that, despite their classification as plankton, closed populations are more common in jellyfish species than previously thought, and that the distances separating closed jellyfish populations can be surprisingly small. The Magnetic Island *C. sivickisi* stock was separated from adjacent mainland populations by at least 8 km. Comparable scales of separation have been reported between stocks of the cubozoan *Chironex fleckeri*^5,15,16^ and between stocks of the scyphozoans *Aurelia aurita*^24^, *Cassiopea* spp.^44^, *Catostylus mosaicus*^23,45^ and *Mastigias papua*^46^. All the listed species have bipartite lifecycles. Some of the species also inhabit enclosed or semi-enclosed environments (e.g., marine lakes and estuarine bays) and have strong swimming medusae.

The predicted limited spatial scales of ecological connectivity from the Magnetic Island *C. sivickisi* population contradict with the currently established cosmopolitan distribution of *C. sivickisi*. *C. sivickisi* are found across the Pacific and in the Indian and Atlantic Oceans^6,17–19^. It is highly likely that lineages within this distribution have been isolated by vicariance events and have diverged from a common ancestor into incipient (morphologically similar and can interbreed) or cryptic (morphologically similar but cannot interbreed) species. Ancient vicariance events have been put forward as the most likely mode of diversification in Cubozoa that inhabit nearshore estuarine and/or coastal habitats and that are, therefore, unlikely to cross large bodies of open water^47^.

## Conclusions

*C. sivickisi* medusae do not wander or drift as for other plankton. The medusae can restrict their distribution to narrow bands of fringing reef by selectively attaching to the reef to avoid dispersive flows. The population of *C. sivickisi* on Magnetic Island extended along the entire east coast, and likely represented a robust, ecological distinct stock with bay-scale (hundreds of metres to kilometres) substructure. There was limited potential for medusae from Magnetic Island to connect with mainland populations over 8 km away, either directly or via Middle Reef. The small scales of connectivity simulated in the Magnetic Island population suggest that genetic heterogeneity may be common in *C. sivickisi* populations at surprisingly small spatial scales. We predict that incipient or cryptic species may be found within the cosmopolitan distribution of *C. sivickisi*.

## Supplementary Information

Supplementary Information.
