# Supplementary material for: Behavioural and oceanographic isolation of an island-based jellyfish (Copula sivickisi, Class Cubozoa) population
Source: Sci Rep. 2021 May 13;11:10280. doi: 10.1038/s41598-021-89755-7 (PMC8119444; doi:10.1038/s41598-021-89755-7)
Supplement: Supplementary file 1 — Supplementary Information. [file 41598_2021_89755_MOESM1_ESM.docx]

Behavioural and oceanographic isolation of an island-based jellyfish (*Copula sivickisi*, Class Cubozoa) population

Supplementary information

*Jodie A. Schlaefer^1,2,3^, Eric Wolanski^2,4^, Jonathan Lambrechts^5^, Michael J. Kingsford^2,3^

^1^Research Hub for Coral Reef Ecosystem Functions, Townsville, QLD 4811, AUS

^2^College of Science and Engineering, James Cook University, Townsville, QLD 4814, AUS

^3^ARC Centre of Excellence for Coral Reef Studies, Townsville, QLD 4814, AUS

^4^TropWATER, James Cook University, Townsville, QLD 4814, AUS

^5^Institute of Mechanics, Materials and Civil Engineering, Université de Louvain, Louvain-La-Neuve, 1348, Belgium

*Correspondence to jodie.schlaefer@my.jcu.edu.au

**Biophysical model setup and validation**

# HYDRODYNAMIC MODEL

The currents around Magnetic Island and in the Townsville region (Queensland, Australia) were simulated by the Second-generation Louvain-la-Neuve Ice-ocean Model (SLIM; Fig. S1; [1]). The two-dimensional depth averaged version of SLIM was used because the waters of interest were shallow (< 20 m deep) and vertically well mixed, like other waters in the Great Barrier Reef (GBR) lagoon [2]. The two-dimensional version of SLIM has previously been used to accurately simulate the hydrodynamics of shallow systems that are well mixed [1] and partially mixed [3]. In SLIM, the shallow-water equations are discretised and solved in space with a second order discontinuous Gerlerkin finite element method and in time with a second order implicit Runge-Kutta method [1]. Further, the dissipation due to bottom friction was calculated with a Chezy-Manning scheme, and the bottom friction was calculated according to the Chezy-Manning-Strickler formulation. The turbulent velocity was calculated with a Smagorinsky scheme. The unstructured SLIM grid was composed of 74, 438 triangles and the side lengths of the triangles ranged from 30 m to 7.5 km. There were, therefore, 223,314 (74, 438 × 3) degrees of freedom by field (sea surface elevation, and zonal and meridional current components).

The model was forced with tides and currents at the open boundary, and with wind over the entire domain. The values of these forcings were derived from eReefs GBR 4, a heavily cross-checked regional scale model of the hydrodynamics of the GBR system and the adjoining Coral Sea [4]. The introduction of the open boundary forcings was buffered by situating the model boundary 10 km from the 200 m isobath and setting all depths in the model bathymetry > 200 m to 200 m. All inputs into the model were additionally buffered by ramping them up over a period of 48-hours. The model bathymetry was derived from an open source high resolution (30 m) depth model of the GBR [5]. The high-resolution bathymetry was averaged/smoothed to a 100 m resolution with QGIS (version 2.18.16). Erroneous deep holes interspersed near shore waters in [5], and these holes had to be corrected before the smoothed bathymetry could be used in the SLIM model (Fig. S2).


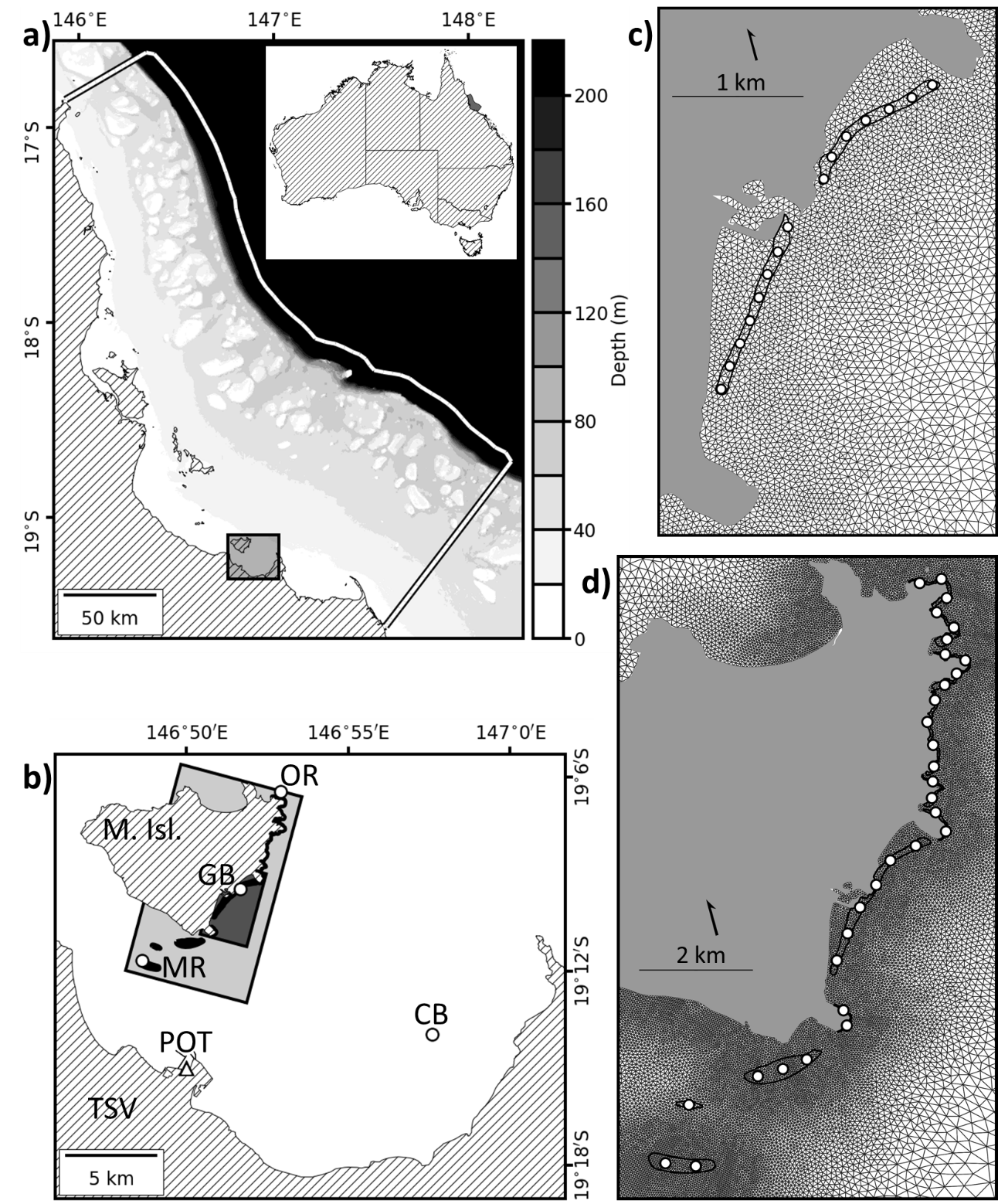


Fig. S1. Biophysical model development, validation and application. a) The model domain (white line with black border), and the bathymetry of the modelled region. The color bar indicates the depth (m). Depths > 200 m are shown in black. The inset shows the location of the model domain (grey polygon) in Queensland, and its scale in relation to Australia. The location of pane b is indicated by the grey box. b) The instruments used to validate the hydrodnamic simulations in the Townsville (TSV)/Magnetic Island (M. Isl.) region. The white dots mark the locations of the current meters at Cleveland Bay (CB), Middle Reef (MR), Geoffrey Bay (GB) and Orchard Rocks (OR). The white triangle marks the location of the tide gauge at the Port of Townsville (POT). The locations of panes c and d are indicated by the dark and light grey boxes respectively. Reefs from OR to MR are shown in black. c) and d) The seed locations (white) and reefal habitats (black outline) used in the role of behaviour in retention and population structure analyses respectively. The variable resolution SLIM mesh is shown in c and d. Land is filled with a hatch pattern in panes a and b, and filled grey in panes c and d.

## Bathymetry correction

The bathymetry raster was corrected in a stepwise process. Firstly, the raster pixels that made up the holes were visually located in QGIS and marked using the serval plugin (version 0.8.1; Fig. S2b). The raster was then modified in python (version 3.6.6). The marked holes were evaluated one line at a time along a predefined direction (i.e., along the west to east horizontal, along the north to south vertical, along the north west to south east diagonal or along the north east to south west diagonal). If the line was adjoined by water on one or both sides in the direction of interest it was filled using the linspace function in the python module numpy (version 1.13.3). Each hole pixel was also assigned a weighting based on its proximity to the water pixels/pixel that were used to generate the linspace fill sequence. This process was repeated in different directions until all hole identified pixels were filled. Four corrected bathymetry variants were generated, differing by the directions the lines were filled in first. The variants were averaged based on the weightings assigned to the filled raster pixels. The final corrected bathymetry was generated by smoothing the averaged bathymetry with a gaussian filter (gaussian blur function in python module cv2 version 3.2.0; Fig. S2c).


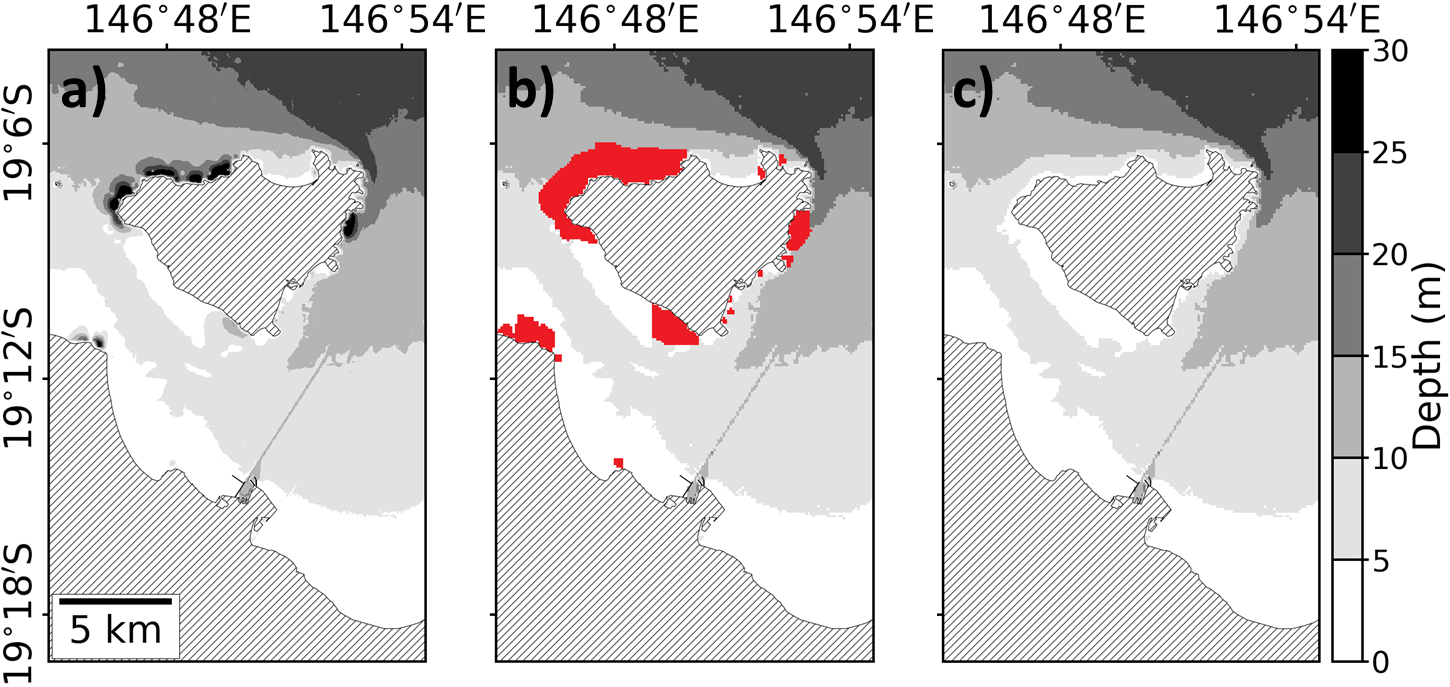


Fig. S2. The bathymetry correction in the Townsville/Magnetic Island region, Queensland, Australia. a) the raw bathymetry from [5] with visable nearshore holes. b) the identified holes (red area). c) the corrected bathymetry. The color bar indicates the depth (m). Depths > 25 m are shown in black. Land is filled in a hatch pattern.

## Validation

The tide and currents simulated by SLIM were validated against measured tide and current data. Open source tidal data were procured from Maritime Safety Queensland (Queensland Government). Data from drag-tilt current meters were sourced from the Australian Government's National Environmental Science Program - Tropical Water Quality Hub Project 2.1.5 (<https://nesptropical.edu.au/index.php/round-2-projects/project-2-1-5/>). Specifically, we compared the measured and simulated: 1) tidal anomalies at the Port of Townsville, and 2) zonal (west to east) and meridional (south to north) current components at Geoffrey Bay, Middle Reef, Orchard Rocks and Cleveland Bay (Fig. S1b). These comparisons were made over the periods of both the 2016 role of behaviour in retention analysis (one month, 17 September to 17 October 2016) and the 2017 population structure analysis (four months, 2 September to 28 December 2017). The SLIM outputs were modelled as linear functions of the corresponding measurements in linear regression analyses to quantify how closely the simulated data matched the measured data. The regressions were performed using the statsmodels (version 0.10.1) python package. All x-y plots of the simulated and measured data sets showed linear relationships with approximately elliptical clouds of data. This indicated that the regression assumptions of linearity, homogeneity, and normally distributed residuals were met. Notably, drag-tilt current meters can incorrectly measure the current in wave dominated flows (e.g. [6, 7]). This could have contributed to any mismatches between the measured and simulated currents, particularly as flows become more wave dominated in high wind conditions.

SLIM accurately reproduced the tides in the Magnetic Island region (Fig. S1b, Table S1, Fig. S3ab). The tidal anomalies simulated at the Port of Townsville in the 2016 role of behaviour in retention analysis and in the 2017 population structure analysis both closely matched the anomalies measured at the port over the periods of the analyses (2016: Fig. S3a and Fig. S3b [y ~ x]; 2016 and 2017: m ~ 1, c ~ 0 and RMSE ~ 0).

The currents simulated at Magnetic Island and in the surrounding region matched current meter measurements (Fig. S1b, Table S1, Fig. S3cdef). The currents simulated at Geoffrey Bay were well represented in SLIM. Although the west-east (U) and south-north (V) components of the currents simulated in 2016 tended to be greater than the measured components, the trends were captured well (Fig. S3cd, RMSE range: 0.24 to 0.26). As the flood (negative U and V) and ebb (positive U and V) tidal current peaks were both slightly overestimated, the simulated net tidal transport would have closely matched the real tidal transport. The measured trends in the U and V current components at Geoffrey Bay were captured equally well in SLIM in 2017 (RMSE range: 0.23 to 0.27); although, U went from being slight overestimated to being slightly underestimated (m < 1). The simulated U component at Middle Reef was somewhat inflated in both 2016 and 2017 (m > 1), and the V component was generally weaker than measured (m < 1); however, the trends were captured effectively (RMSE range: 0.16 to 0.26). At Orchard Rocks, the U and V current components were both slightly underestimated in 2016 (m < 1) but the overall fit was good (RMSE range: 0.13 to 0.15). The currents measured at a site in Cleveland Bay were well represented in the model (RMSE range: 0.11 to 0.24); although, the U component could be underestimated (m < 1) and the V component could be overestimated (m > 1).

Table S1. SLIM validation. SLIM data have been compared with measured data (Fig. S1b) over the one-month period of the role of behaviour in retention analysis (17 September to 17 October **2016**) and over the four-month period of the population structure analysis (2 September to 28 December **2017**). Metrics of the accuracy of the tidal anomaly (**TA**) simulated at the Port of Townsville (**POT**) and of the currents simulated at Geoffrey Bay (**GB**), Middle Reef (**MR**), Orchard Rocks (**OR**) and Cleveland Bay (**CB**) are presented. The currents have been broken up into their zonal (**U**, west to east) and meridional (**V**, south to north) components. Linear regression lines of the form y = mx + c have been generated, where the SLIM outputs (y) are modelled as a function of the measured values (x). **m** is the slope of the regression line and **c** is the y-axis intercept. In a perfect model m = 1 and c = 0. The normalised root mean square error (**RMSE**, i.e. the standard deviation of the residuals) has also been presented for each regression line. The closer the RMSE is to 0, the better the fit of the SLIM output with the corresponding measured data. The number of measurements taken by the instruments during each period are indicated (**n**). Where cells are blacked out, no measured data were available.

|  | | **2016** | | | | **2017** | | | |
| --- | --- | --- | --- | --- | --- | --- | --- | --- | --- |
|  |  | **m** | **c** | **RMSE** | **n** | **m** | **c** | **RMSE** | **n** |
| **TA** | **POT** | 1.03 | -0.04 | 0.03 | 4321 | 1.03 | -0.11 | 0.04 | 13530 |
| **U** | **GB** | 1.39 | 1.05x10^-3^ | 0.26 | 1859 | 0.87 | 0.02 | 0.27 | 13766 |
|  | **MR** | 1.34 | 0.01 | 0.20 | 4321 | 2.00 | -0.04 | 0.26 | 13587 |
|  | **OR** | 0.59 | -2.75x10^-3^ | 0.15 | 4321 |  | | | |
|  | **CB** | 0.54 | -8.79x10^-3^ | 0.15 | 4321 | 0.59 | -4.04x10^-3^ | 0.11 | 5689 |
| **V** | **GB** | 1.13 | 0.04 | 0.24 | 1859 | 1.21 | 0.02 | 0.23 | 13766 |
|  | **MR** | 0.60 | 0.01 | 0.16 | 4321 | 0.60 | 0.01 | 0.17 | 13587 |
|  | **OR** | 0.61 | -7.96x10^-4^ | 0.13 | 4321 |  | | | |
|  | **CB** | 1.32 | 0.01 | 0.24 | 4321 | 1.21 | 0.02 | 0.15 | 5689 |


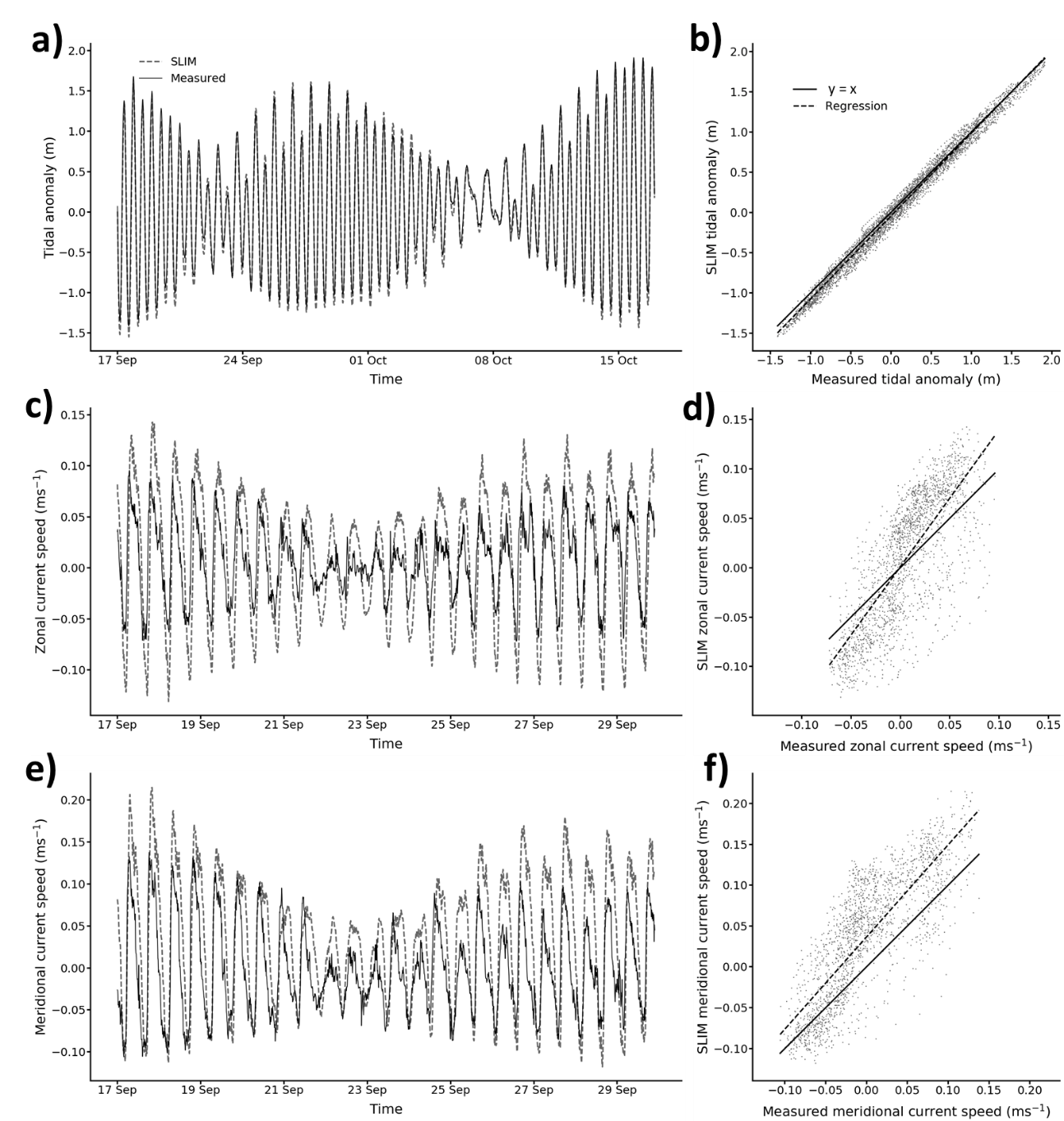


Fig. S3. Visualisation of the hydrodynamic model validation. a) time series and b) x-y plot comparing the measured and simulated tidal anomalies at the Port of Townsville. Data are shown for the full period of the role of behaviour in retention analysis (17 September to 17 October 2016). c) time series and d) x-y plot comparing the measured and simulated zonal (west to east) components of the currents at Geoffrey Bay. Data are shown for the period of the role of behaviour in retention analysis for which current meter data were available (17 September to 30 September 2016). e) time series and f) x-y plot comparing the measured and simulated meridional (south to north) components of the currents at Geoffrey Bay. Data are shown over the same time period as the zonal components. In all time series, the measured data are shown with a solid black line, and the SLIM data are shown with a dashed grey line. In all x-y plots, the regression line of the SLIM data (y) modelled as a function of the measured data (x) is shown as a black dashed line and compared to a solid black identity line (y = x). The locations of the measuring instruments are shown in Fig. S1b.

# BEHAVIOURAL MODEL

The movements of the *Copula sivickisi* medusae were modelled as a function of three vectors: 1) the current at their location as determined by the described hydrodynamic model, 2) the horizontal diffusivity ($K_{h}$) from sub-mesh scale turbulent mixing, and 3) the space and time dependent programmed behaviour of the medusae.

## Horizontal diffusivity

An Okubo scheme [8] was used to model $K_{h}$ as a function of the local mesh size ($l$) following de Brye et al. [9]:

$K_{h}= \alpha\times l^{1.15}$ (1)

The value of the coefficient α was set to 2 × 10^-4^ m^0.85^ s^-1^, derived directly from [8]. The dispersal of passive particles released from a single location in Nelly Bay, Magnetic Island was analysed through time to determine the resulting effective diffusivity (combined sub-mesh and mesh scale diffusivity). Measures of the diameter of the passive particle plume were taken as the plume spread from the initial seed location. The effective diffusivity was estimated to be < 20 m^2^ s^-1^. Appropriately, this is less than the value of 25 m^2^ s^-1^ that Hrycik et al. [10] calculated for the more exposed Northumberland Strait, Canada.

## *C. sivickisi* medusae behaviour

The behaviour of the *C. sivickisi* medusae was programmed to change with their proximity to reefal habitat (on/off) and the time of day (day/night). The extent of the reefal habitat was determined from historic satellite images sourced from Google Earth (version 7.3.2.5491). Where possible, the presence of reefal habitat was later validated with JCam footage (present study; [11]). Medusae were classified as ‘on habitat’ if they lay within 100 m of the midlines of the historic habitat bands and were classified as ‘off habitat’ if they were greater than 100 m from the midlines. The *C. sivickisi* medusae were programmed to be nocturnal, so they were inactive during the day and active at night [11, 12]. Between 07:00 and 18:57 was considered day time and between 19:00 and 06:57 was considered night time, corresponding to the normal time of sunrise and sunset in the region during the *C. sivickisi* season (September to November).

A ‘base’ candidate model of the behaviour of *C. sivickisi* medusae was initially developed, and this base model was modified to generate the ‘dependent’ candidate model. The two models had the same off habitat behaviour; medusae off habitat had no directional swimming cues, and this was consistent across day and night hours (Table S2). The two models also had the same on habitat, daytime behaviour; medusae on habitat during the day attached themselves to the habitat and were, therefore, not affected by the current. The models differed in the programmed on habitat, night time behaviour. Base model medusae on habitat at night swam toward the habitat midline in all current speeds. In contrast, dependent model medusae on habitat at night would only swim if the current speed at their position was less than a set cut off. Medusae in current speeds greater than or equal to the cut off fixed their positions by attaching to habitat. In both candidate behavioural models, and in all times and positions, medusae unattached to habitat were assumed to stay close to the bottom of the water column. Further, the surface currents were assumed to be faster than the currents near the bottom (current shear), and so only half of the depth averaged current velocities simulated in SLIM were applied to near bottom medusae. The inclusion of each component of the behavioural models is justified in detail in Table S2.

Table S2. Descriptions of the behaviours included in the Base (B) and Dependent (D) models of *Copula sivickisi* medusae behaviour, accompanied by justifications for their inclusion.

| Description of modelled behaviour | Model | Justification **[reference]** |
| --- | --- | --- |
| Nocturnal | B & D | In tank experiments, the level of activity in *C. sivickisi* medusae increased from day to night, coinciding with a reduction in their level of inactivity **[11, 12].** Further, *C. sivickisi* medusae have been sampled with plankton nets during the day and at night in their natural environment and have been almost entirely absent from the daytime samples **[11, 12]** |
| ‘On habitat’ within 100 m of the habitat midline and ‘off habitat’ beyond this zone | B & D | The distribution of *C. sivickisi* medusae was mapped at a fine spatial scale in Nelly Bay and Geoffrey Bay at Magnetic Island. Medusae were most abundant at sites on a dense band of fringing reef habitat dominated by *Sargassum sp.* algae and coral. Medusae were also abundant at sites with high to moderate habitat availability, within 110 m of the dense band **[11]** |
| Swim to habitat midline | B & D | *C. sivickisi* medusae displayed a strong preference for *Sargassum* over other available habitats in a habitat choice experiment **[11]**. In another experiment, *C. sivickisi* medusae selectively attached to different habitats including the underside of a coral **[12]** |
| Attach to habitat (daytime) | B & D | A single medusa was filmed attaching to *Sargassum* in its natural environment **[11]**. Numerous *C. sivickisi* medusae were observed attaching to *Sargassum* **[11]** and coral **[12]** in habitat choice experiments |

Table S2. Continued.

| Description of modelled behaviour | Model | Justification **[reference]** |
| --- | --- | --- |
| a) Maintain positions near the bottom of the water column and, thereby,  b) experience a reduced current speed, estimated at half of the depth average current speed simulated in SLIM | B & D | a)In surface and near bottom plankton tows conducted both during the day and at night, *C. sivickisi* medusae were almost exclusively collected in the near bottom plankton tows at night **[11]**. Further, the proclivity of medusae to stay near the bottom was so strong that their distribution was found to be depth stratified even in the very shallow system sampled by **[11]**. Specifically, over half (29 of 46, 63%) of the tows were conducted in waters less than 3.5 m deep, where < 1 m would have separated the surface and bottom samples (Fig. S4)  b) Current speeds can tend toward zero with increasing proximity to the bottom **[13, 14]** |
| Attach to habitat (night time) when the current speed exceeds a set cut off. A range of cut-offs were modelled (3, 4.5, 6, 7.5 and 9 cm s^-1^) | D only | In swim trials with stepwise increasing currents, 17 of 41 medusae (more than 40%) attached to the side of the tank to avoid being pushed back by the current. Thirteen attached in slow currents ≤ 3 cm s^-1^ and the remaining four attached in moderate currents ≥ 6 cm s^-1^ **[11]**  The range of modelled cut-offs covered and extended the range of current speeds at which *C. sivickisi* medusae attached to the tank in the swim trials of **[11]** |


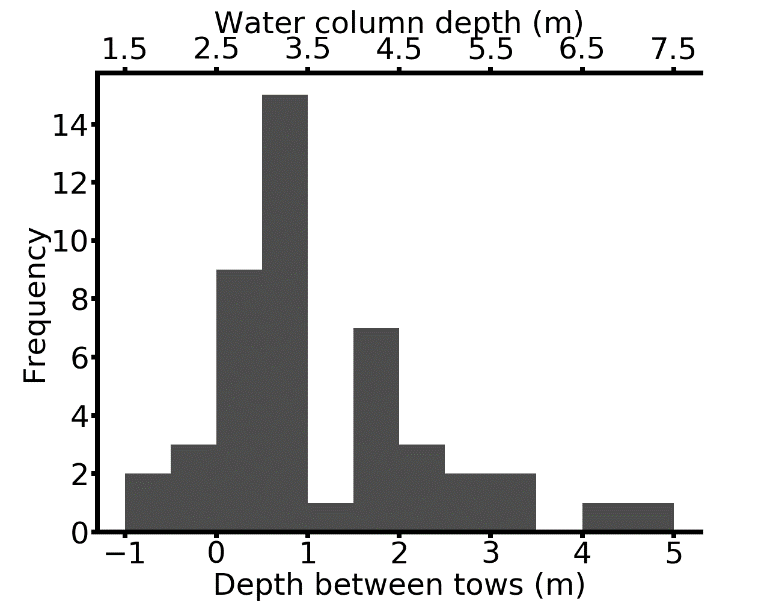


Fig. S4. Frequency distribution of the water column depths (D_wc_), and related theoretical ‘depth between the surface and bottom tows’ for the plankton tows conducted in [11]. The between tow depths were calculated as: D_wc_ – 2 × ND – 1. Two times the net diameter (ND) was subtracted to account for the depth covered in the surface and bottom tows. An additional 1 m was subtracted as bottom tows were performed 1 m from the bottom. Note the surface and bottom tows were not done consecutively, so sampled areas did not physically overlap where negative theoretical between depths were calculated. n = 46.

## Run details

Table S3. Detailed specifications of the model runs that constituted the primary role of behaviour in retention analysis and the sensitivity analysis, and justifications for their form and application. B = Base, D = Dependent and P = Passive.

| Role of behaviour in retention |  | |
| --- | --- | --- |
| Specification | Details | Justification **[reference]** |
| Candidate models (n) | B, D and P (5 replicate runs per candidate model) | 5 runs were sufficient to determine the inter-run variability **[15]** |
| Release date | A date in September, near the start of the 2016 *C. sivickisi* medusae season (day/month): 17/9 | Medusae were released into seasonally appropriate currents |
| Seed locations | 15 seed locations spaced at 200 m intervals along the bands of fringing reef in Nelly Bay and Geoffrey Bay (Fig. S1c) | The spatial extent of the seed locations covered the known distribution of *C. sivickisi* medusae in Nelly Bay and Geoffrey Bay **[11]** |
| Number of medusae released per location | 1000 per location; 15,000 in total  Released simultaneously at mid night | The same trends were simulated in the replicate model runs, suggesting enough particles were released to sufficiently capture the variability in the model system **[15]** |
| Mortality | No mortality | No mortality was included as these short runs were focused on projecting the level of retention |
| Duration | 30 days | Approximately the length of one lunar cycle, allowing for the assessment of retention under spring and neap tides |

Table S4. Detailed specifications of the model runs that constituted the population structure analysis, and justifications for their form and application. D = Dependent.

| Population structure |  | |
| --- | --- | --- |
| Specification | Details | Justification **[reference]** |
| Candidate model (n) | D (5 replicate runs) | 5 runs were sufficient to determine the inter-run variability **[15]** |
| Release dates (n) | The five nights leading up to and including each of the 5 New (N) or Full (F) moons within the 2017 *C. sivickisi* season (day/month):  2/9, 3/9, 4/9, 5/9, 6/9 (F), 16/9, 17/9, 18/9, 19/9, 20/9 (N), 2/10, 3/10, 4/10, 5/10, 6/10 (F), 16/10, 17/10, 18/10, 19/10, 20/10 (N), 31/10, 1/11, 2/11, 3/11, 4/11 (F)  (25 nights) | Juvenile *C. sivickisi* medusae are generally present at Magnetic Island from the start of September to the start of November **[unpublished data]**  Tidal currents tend to amplify in strength in the lead up to new and full moons. Medusae were, therefore, released in a range of tidal conditions. |
| Seed locations | 32 seed locations spaced approximately every 500 to 600 m along the near continuous band of reefal habitat which runs from Middle Reef, along the eastern coast of Magnetic Island to Florence Bay (Fig. S1d) | The spatial extent of the seed locations covered the known distribution of *C. sivickisi* medusae on the eastern coast of Magnetic Island, as determined from the JCam survey in the **present study** |
| Number of medusae released per location | 2,400 per location, per release date; 1,920,000 in total  Released 200 at a time, on the hour over 12 night-time hours from 19:00 to 06:00 | The same trends were simulated in the replicate model runs, suggesting enough particles were released to sufficiently capture the variability in the model system **[15]**  Hourly releases ensured medusae were released in all states of the tide following **Grech et al. [16]** |

Table S4. Continued.

| Mortality | Natural mortality:  $N_{t}= N_{0} \times e^{-z\times t}$  Where the number of medusae alive at time t (N_t_) is a function of the initial number of medusae (N_0_) and the instantaneous mortality rate (z = 0.135 day^-1^)  All medusae remaining after 54 days were killed | Natural mortality **[17]** was added to simulate the exponential attrition that would likely occur throughout the medusae season (Fig. S5b).  z was calculated from a catch curve of *C. sivickisi* medusae **[unpublished data]** following **[17, Fig. S5a]**. See ‘Simulated mortality’ section  54 days was near the maximum age of 56 days for *C. sivickisi* medusae, determined from statolith ring counts **[unpublished data]** following **Gordon et al. [18]** and only a small proportion of animals will survive to a species maximum age |
| --- | --- | --- |
| Duration | 117 days | The time between the first date medusae were released (2/9/2017) and last date the movements of medusae were simulated (i.e. 28/12/2017, 54 days from the last date of release [4/11/2017]) |

## Simulated mortality

The daily instantaneous mortality rate of *C. sivickisi* medusae was estimated from a catch curve following established methods. The catch curve was built from 211 medusae all caught from Geoffrey Bay, Magnetic Island on 30 October 2017 (Haack pers. comm.). The caught medusae were sized and the medusae were counted in different 0.25 mm size bins (Haack pers. comm.). The size bins were then converted to age bins based on the linear relationship between medusae size and the number of rings on their statoliths (Haack pers. comm.). Rings were assumed to be laid down daily following [18]. From this data and following [17], we log_e_ transformed the frequency of the different age bins. The transformed frequency was then modelled as a linear function of the corresponding age bin midpoint in a regression analysis (python 3.7.4, statsmodels 0.11.0, Fig. S5a). The data points to the left of the peak in frequency were not included in the regression, as suggested by [17]. The slope of the regression line (0.135) was taken as the daily instantaneous mortality rate of *C. sivickisi* medusae. The rate was then used to populate a model of natural mortality within the biophysical model (Table S4). The natural mortality was simulated as exponential decay, with a curve that approached but never reached 100% mortality (i.e. no medusae remaining in the biophysical model, Fig. S5b). The population structure model runs were terminated at 54 days, so the small percentage of model medusae still alive at that time were killed (3.2 % of released medusae).


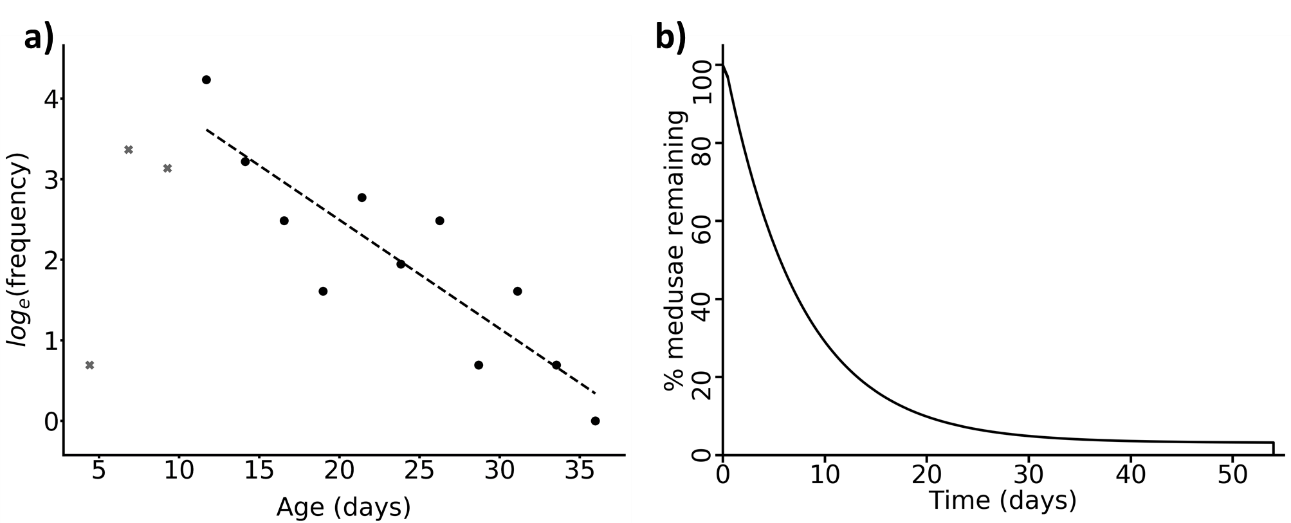


Fig. S5. The simulation of *Copula sivickisi* medusae mortality. a) daily instantaneous mortality rate calculation. The linear regression of the age bin mid-points (x) and the corresponding log_e_ transformed medusae frequencies (y). The points shown with black dots were included in the regression, and the points shown with grey crosses (i.e. points to the left of the peak in frequency [17]) were not. The slope of the regression line (dashed line) was taken as the daily instantaneous mortality rate. b) The shape of the modelled mortality curve showing the exponential decay of *C. sivickisi* medusae numbers through time.

**Sensitivity analysis supplementary results**


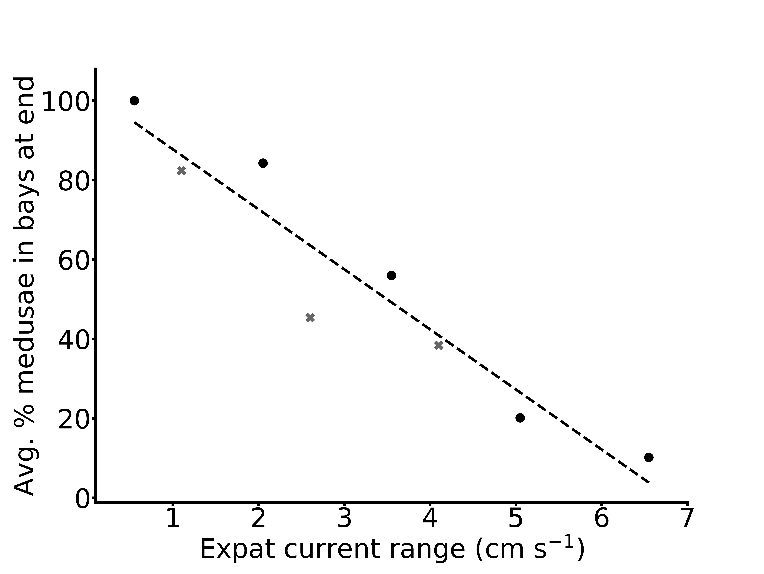


Fig. S6. Supplementary sensitivity analysis results. The linear regression of the range of current speeds with the potential to expatriate the model *C. sivickisi* medusae from reefal habitat (explanatory variable, x) and the average percentage of medusae remaining in Nelly Bay and Geoffrey Bay at the end of the 30 day model runs (y). The size of the expatriating current range was calculated as the speed difference between the medusae swim speed and the set current speed cut-off at which medusae attached to habitat. The black dots show the results for the sensitivity analysis runs where medusae swam at U_sust_ and the grey crosses show the results for runs where medusae swam at U_crit_. The dashed line is the regression line.

**Replicate connectivity matrices**

There was little variability in the inter bay/reef connectivity simulated in the replicate population structure analysis model runs (Fig. S7). The matrices from the replicate runs similarly show: 1) high in-zone retention of *Copula sivickisi* medusae in the bays on the east coast of Magnetic Island, 2) connectivity with adjacent bays over small distances, 3) negligible export of medusae from the island population to Middle Reef, and 4) limited export in the opposite direction (from Middle Reef to Magnetic Island).


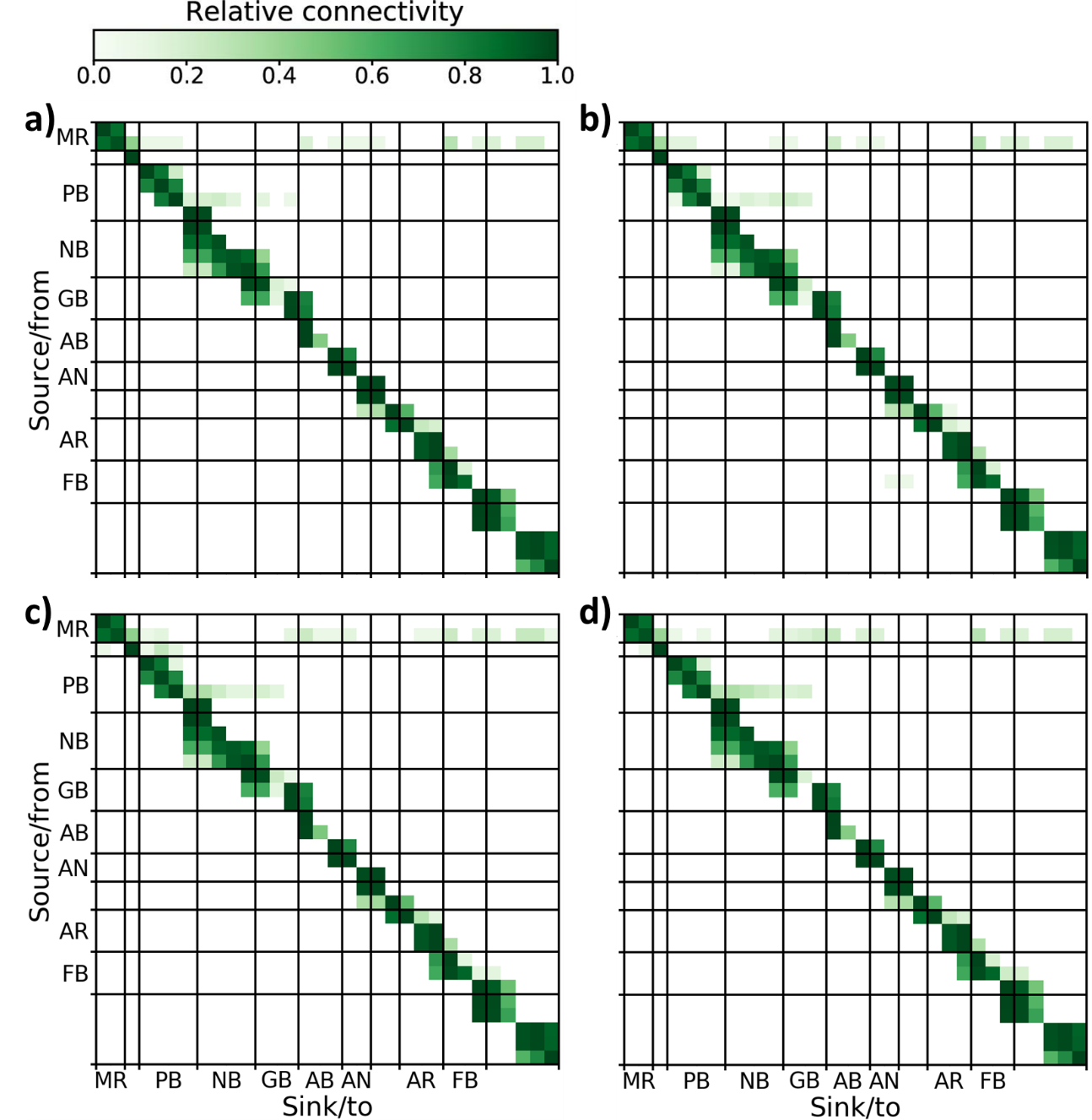


Fig. S7. a) to d) Replicate substructure of the *Copula sivickisi* population at Magnetic Island. The relative connectivity between source/from and sink/to detection zones over the entire 2017 medusae season. The detection zones have been pooled by reefs/bays. Each of the matrices show the results from one of the four replicate model runs not presented in the main text. Five replicate runs were conducted in total. The geographic locations of the reefs/bays are shown in Fig. 1.

# REFERENCES

[1] Lambrechts, J., et al. A multi-scale model of the hydrodynamics of the whole Great Barrier Reef. *Estuar. Coast. Shelf Sci.* **79**, 143-151 (2008).

[2] Luick, J. L., Mason, L., Hardy, T. & Furnas, M. J. Circulation in the Great Barrier Reef Lagoon using numerical tracers and in situ data. *Cont. Shelf Res.* **27**, 757-778 (2007).

[3] Pham Van, C., et al. Simulations of the flow in the Mahakam river–lake–delta system, Indonesia. *Environ. Fluid Mech.* **16**, 603-633 (2016).

[4] Herzfeld, M., et al. eReefs marine modelling: Final report (CSIRO, 2016)

[5] Beaman, R. J. High-resolution depth model for the Great Barrier Reef - 30 m. *Geoscience Australia, James Cook University and the Australian Hydrographic Service* https://doi.org/10.4225/25/5a207b36022d2 (2017).

[6] Hansen, A. B., Carstensen, S., Christensen, D. F. & Aagaard, T. Performance of a tilt current meter in the surf zone. Presented at *Coastal Dynamics* (2017).

[7] Anarde, K. & Figlus, J. Tilt current meters in the surf zone: Benchmarking utility in high-frequency oscillatory flow. Presented at *Coastal Dynamics* (2017).

[8] Okubo, A. Oceanic diffusion diagrams. *Deep-Sea Res.* **18**, 789-802 (1971).

[9] de Brye, B., et al. A finite-element, multi-scale model of the Scheldt tributaries, river, estuary and ROFI. *Coast. Eng.* **57**, 850-863 (2010).

[10] Hrycik, J. M., Chasse, J., Ruddick, B. R. & Taggart, C. T. Dispersal kernel estimation: A comparison of empirical and modelled particle dispersion in a coastal marine system. *Estuar. Coast. Shelf Sci.* **133**, 11-22 (2013).

[11] Schlaefer, J. A., Wolanski, E., Yadav, S. & Kingsford, M. J. Behavioural maintenance of highly localised jellyfish (*Copula sivickisi*, class Cubozoa) populations. *Mar. Biol.* **167**, 40; 10.1007/s00227-020-3646-6 (2020).

[12] Garm, A., Bielecki, J., Petie, R. & Nilsson, D. E. Opposite patterns of diurnal activity in the box jellyfish *Tripedalia cystophora* and *Copula sivickisi*. *Biol. Bull.* **222**, 35-45 (2012).

[13] Davies, A. M. & Lawrence, J. Examining the influence of wind and wind-wave turbulence on tidal currents, using a 3-dimensional hydrodynamic model including wave-current interaction. *J. Phys. Oceanogr.* **24**, 2441-2460 (1994).

[14] Fischer, H. B., List, E. J., Koh, R. C. Y., Imberger, J. & Brooks, N. H. Mixing in Inland and Coastal Waters (Academic Press, Inc., 1979).

[15] Brickman, D. & Smith, P. C. Lagrangian Stochastic Modeling in Coastal Oceanography. *J. Atmos. Ocean. Technol.* **19**, 83-99 (2002).

[16] Grech, A., et al. Spatial patterns of seagrass dispersal and settlement. *Divers. Distrib.* **22**, 1150-1162 (2016).

[17] Ricker, W. E. Computation and interpretation of biological statistics of fish populations. *Bull. Fish. Res. Board Can.* **191**, 1-382 (1975).

[18] Gordon, M., Hatcher, C. & Seymour, J. Growth and age determination of the tropical Australian cubozoan *Chiropsalmus sp.* *Hydrobiologia* **530**, 339-345 (2004).
